# Nonlinear Analysis of Auscultation Signals in TCM Using the Combination of Wavelet Packet Transform and Sample Entropy

**DOI:** 10.1155/2012/247012

**Published:** 2012-05-29

**Authors:** Jian-Jun Yan, Yi-Qin Wang, Rui Guo, Jin-Zhuan Zhou, Hai-Xia Yan, Chun-Ming Xia, Yong Shen

**Affiliations:** ^1^Center for Mechatronics Engineering, East China University of Science and Technology, Shanghai 200237, China; ^2^Syndrome Laboratory of TCM, Shanghai University of Traditional Chinese Medicine, Shanghai 201203, China

## Abstract

Auscultation signals are nonstationary in nature. Wavelet packet transform (WPT) has currently become a very useful tool in analyzing nonstationary signals. Sample entropy (SampEn) has recently been proposed to act as a measurement for quantifying regularity and complexity of time series data. WPT and SampEn were combined in this paper to analyze auscultation signals in traditional Chinese medicine (TCM). SampEns for WPT coefficients were computed to quantify the signals from qi- and yin-deficient, as well as healthy, subjects. The complexity of the signal can be evaluated with this scheme in different time-frequency resolutions. First, the voice signals were decomposed into approximated and detailed WPT coefficients. Then, SampEn values for approximated and detailed coefficients were calculated. Finally, SampEn values with significant differences in the three kinds of samples were chosen as the feature parameters for the support vector machine to identify the three types of auscultation signals. The recognition accuracy rates were higher than 90%.

## 1. Introduction

TCM is considered a unique medical system because of its basic theories describing the physiology and pathology of the human body, disease etiology, diagnosis, and differentiation of symptom complexes. The zang-fu organs, according to TCM theories, comprise the core of the human body as an organic entity in which tissues and sense organs are connected through a network of channels and collaterals (blood vessels). In traditional Chinese medicine the zang and fu organs more importantly represent the generalization of the physiology and pathology of certain systems of the human body instead of simply anatomical substances, but Zang fu is comprised of the five zang and six fu organs. The five zang include heart, liver, spleen, lung, and kidney. The six Fu are the gallbladder, stomach, large intestine, small intestine, bladder, and triple burner. When one falls ill, a dysfunction in the zang-fu organs may be reflected on the body's surface through the channels and their collaterals. At the same time, diseases involving body surface tissues may also affect their related zang or fu organs. Furthermore, the affected zang or fu organs may influence each other through internal connections [1]. In addition, auscultation, one of the auscultation and olfaction methods in TCM diagnosis, is used to detect vocal changes reflecting the functional activities of zang-fu organs and abundance or decline of the qi, blood, and body fluid.

Auscultation was clearly illustrated as early as in the Internal Classic of Huang Di [2], which provided the theoretical basis for clinical diagnosis in terms of listening to the vocal change. However, complete acoustic diagnostic methods have not been formulated. After the Ming and Qing Dynasties, auscultation gradually attracted the attention of the medical field with both theoretical content and clinical application considerably developed. Thus, a considerable distinctive step-by-step diagnostic method was formed. People around the world made substantial progress in the objective research of auscultation in the recent years with the development of computer and signal processing technology.

Mo made a frequency spectral analysis on the voice of cough patients using digital sonograph [3]. Wang and Yan performed a number of studies on the nonlinearity of the vowel /a/ signals of healthy persons and patients with deficiency syndrome by applying delay vector variance [4, 5]. These studies were effective attempts on the objective auscultation research. Chiu et al. proposed four novel acoustic parameters, such as the average number of zero crossings, variations in local peaks and valleys, variations in first and second formant frequencies, and the spectral energy ratio, to analyze and identify the characteristics among non-, qi-, and yin-deficient subjects [6].

There are several other studies on auscultation around the world [7–11]. These methods have provided a good basis for objective auscultation in clinical diagnosis. However, auscultation signal analysis and recognition are still in the initial stage. The experiment are conducted on a small sample database. Thus the recognition is not satisfactory such that further investigation is necessary to be carried out based on these studies.

The variations in energy imply corresponding changes in signal characteristics considering the changes in the normal and abnormal voice signals corresponding with the changes in the spatial distribution of the voice signal energy. In other words, the different signal frequency components can represent the different physical properties of the measured signal [12, 13]. Compared with the traditional Fourier transform time-frequency analytical method, the wavelet transform (WT) can reveal more information on signals based on multiscale and multiresolution decomposition. Wavelet packets have recently been applied to analyse auscultation signals because of their capability of partitioning both low- and high-band frequencies unlike the WT that often fails to capture accurately high-frequency information [14–16].

Both approximate entropy (ApEn) and sample entropy (SampEn) can represent the signal complexity which can be used in many biomedical fields. ApEn was proposed by Pincus and Goldberg [17] to compute the quantitative information for the experimental data. However, there are some weak points in the ApEn computation process because its computation in irregular times is affected by a bias, in addition to the inconsistency of ApEn in some cases. SampEn, compared with ApEn, does not count self-matches and shows better relative consistency and less dependence on data length.

Daubechies 4 (db4) wavelet is selected in this paper as the wavelet packet function to decompose the auscultation signals into 5-level wavelet packet coefficients. Then, SampEn is proposed as a feature parameter extracted from these coefficients to analyze quantitatively the auscultation signals. Furthermore, statistical analysis is conducted to obtain the effective feature parameters with significant differences for the recognition of the voice signals. Finally, these feature values are used as input vectors of the support vector machine (SVM) classifier for automatic identification for qi- and yin-deficient, as well as healthy, subjects.

## 2. Materials and Methods

**Figure 1:**
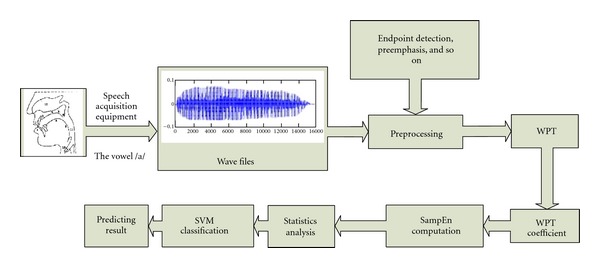
Analytic process of auscultation signals.

Feature parameters of auscultation signals were extracted using a combined WPT and SampEn (Figure 1). Traditional signal processing methods, including the Fourier transform (FT), fast Fourier transform (FFT), and short-time Fourier transform (STFT), cannot reveal the nonlinear information contained in the nonstationary signal. The non-linear information of the auscultation signal can be extracted under different time-frequency resolutions with this scheme.

### 2.1. WPT

Wavelets are generally well crafted to have specific properties that make them available for signal processing. WT has the capability of time-frequency analysis and can draw different frequency bands of the signal. However, with increasing scale, the higher the space resolution ratio of the wavelet functions, the lower the frequency resolution ratio will be. This phenomenon is a drawback of the wavelet function. WPT was developed to adapt the underlying wavelet bases to the contents of a signal. The basic idea is to allow subband decomposition to select adaptively the best basis for a particular signal. The WPT characteristic of narrowing wide window of frequency spectrum with increasing scale overcomes the shortcoming of the WT.

**Figure 2:**
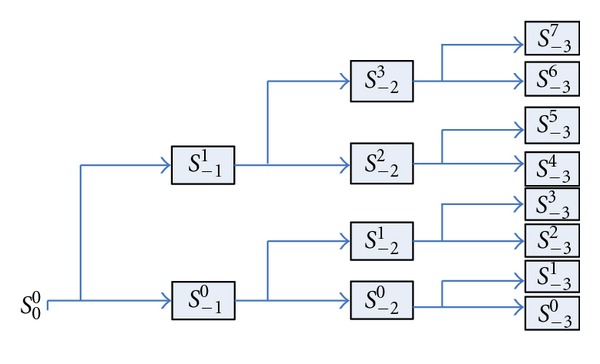
Wavelet packet decomposition tree.

Given a finite energy signal whose scaling space is assumed as *S*_0_^0^, WPT can decompose *S*_0_^0^ into small subspaces *S*_*j*_^*n*^ in a dichotomous way (Figure 2). 

*S*_*j*_^*n*−1^ shows the *n*th subspace in the *j*th resolution level.

The dichotomous way is realised by the following recursive scheme:

(1) $$S_{j+1}^{n}=S_{j}^{2n}⊕S_{j}^{2n+1},\;j\in Z;n\in Z_{+},$$

where *j* ≤ 0 is the resolution level and ⊕ denotes orthogonal decomposition. *S*_*j*+1_^*n*^, *S*_*j*_^2*n*^, and *S*_*j*_^2*n*+1^ are three close spaces corresponding to *S*_*n*_(*t*), *S*_2*n*_(*t*), and *S*_2*n*+1_(*t*), respectively. *S*_*n*_(*t*) satisfies the following equations:

(2) $$\begin{array}{c} S_{2n}\left(t\right)=\sqrt{2}\underset{k\in Z}{\sum }h\left(k\right)S_{n}\left(2t-k\right),\\ S_{2n+1}\left(t\right)=\sqrt{2}\underset{k\in Z}{\sum }g\left(k\right)S_{n}\left(2t-k\right), \end{array}$$

where *h*(*k*) and *g*(*k*) are the coefficients of the low- and the high-pass filters, respectively. The sequence of function {*S*_*n*_}(*n* = 0, 1,…, *∞*) generated from a given function *S*_0_ is called the wavelet packet basis function.

The voice signal is a kind of transient, non-stationary, and random signal. Therefore, db wavelets have been widely implemented because of their advantage in matching the transient components in voice signals. Moreover, another main issue in wavelet analysis is the vanishing moment determined by trial-and-error methods. More points that can be neglected will emerge in the high frequencies if the degree of vanishing moment increases. Therefore, db wavelets with vanishing moments of 4, 6, 8, and 10 were chosen to decompose and reconstitute the voice signals in this study. The db4 wavelet function was selected after analysing the different effects of the wavelet functions to decompose and reconstitute the voice signals because the rate of decay and less point can be neglected. 

The signal is decomposed into two subbands in the first level, namely, low- and high-frequency sub-bands. Then, the low-frequency subbands are further decomposed into lower- and higher-frequency parts in the following level, which was also performed in the high-frequency sub-bands. The same decomposition goes on repeatedly. Then, frequency sub-bands can be partitioned to be consistent with the signal features.

### 2.2. SampEn

SampEn examines time series for similar epochs and assigns a nonnegative number to the sequence, with larger values corresponding to greater complexity or irregularity in the data [18]. Self-matches in the SampEn algorithm are not included in calculating the probability, in contrast to the ApEn algorithm. The time series and similar patterns in parameter *m* and tolerance window *r* are used as two input parameters, which must be set before computation. For a time series *x*(*n*), *N* is the length of the time series. SampEn (*m*, *r*, *N*) is computed as follows [18].

(1) The *m* vectors *X*_*m*_(1),…, *X*_*m*_(*N* − *m* + 1) defined by *X*_*m*_(*i*) = [*x*(*i*), *x*(*i* + 1),…, *x*(*i* + *m* − 1)], for 1 ≤ *i* ≤ (*N* − *m* + 1), are formed. These vectors represent *m* consecutive *x* values starting with the *i*th point.
(2) The distance between vectors *X*_*m*_(*i*) and *X*_*m*_(*j*), *d*[*X*_*m*_(*i*), *X*_*m*_(*j*)], as the absolute maximum difference between their components is defined:
  (3) $$d\left[X_{m}\left(i\right),X_{m}\left(j\right)\right]=\underset{k=0,\ldots ,m-1}{\max}\left(\left|x\left(i+k\right)-x\left(j+k\right)\right|\right).$$
(3) For a given *X*_*m*_(*i*), the number of *j* (1 ≤ *j* ≤ *N* − *m*, *j* ≠ *i*), denoted as *B*_*i*_, is counted such that the distance between *X*_*m*_(*i*) and *X*_*m*_(*j*) is less than or equal to *r*. Then, for 1 ≤ *j* ≤ *N* − *m*,
  (4) $$B_{i}^{m}\left(r\right)=\frac{1}{N-m-1}B_{i}.$$
(4) *B*_*i*_^*m*^(*r*) is defined as
  (5) $$B^{m}\left(r\right)=\frac{1}{N-m}\overset{N-m}{\underset{i=1}{\sum }}B_{i}^{m}\left(r\right).$$
(5) The dimension is increased to *m* + 1, and *B* ^*m*+!^(*r*) was calculated.

Thus, *B* ^*m*^(*r*)  is the probability that two sequences will match *m* points, whereas *B* ^*m*+!^(*r*) is the probability that two sequences will match *m* + 1 points. Finally, SampEn can be defined as

(6) $$\begin{array}{c} \text{SampEn}\left(m,r,N\right)=\underset{N\to \infty }{\sum }-\ln\left[\frac{B^{m+1}\left(r\right)}{B^{m}\left(r\right)}\right] \end{array}.$$

This value is estimated by the statistics:

(7) $$\text{SampEn}\left(m,r,N\right)=-\ln\left[\frac{B^{m+1}\left(r\right)}{B^{m}\left(r\right)}\right].$$

### 2.3. SVM

SVM is a useful machine learning technique that has been successfully applied in the classification area. Classifying data is a common task in machine learning. In most cases, the data to be classified is linearly non-separable but nonlinearly separable in which the nonlinear support vector classifier can then be used. The main idea is to transform the original data into a high-dimensional feature space. Thus, it may be nonlinear in the original input space even though the classifier is a hyperplane in the high-dimensional feature space [19].

The product (*x*, *y*) is replaced by a kernel function *K*(*x*, *y*) to construct a nonlinear support vector classifier. The following are some commonly used kernel functions:

polynomial (homogenous)

(8) $$\begin{array}{c} k\left(x,x^{\prime }\right)={\left(x\cdot x^{\prime }\right)}^{d}, \end{array}$$

polynomial (inhomogeneous)

(9) $$\begin{array}{c} k\left(x,x^{\prime }\right)={\left(x\cdot x^{\prime }+1\right)}^{d}, \end{array}$$

radial basis function

(10) $$\begin{array}{c} k\left(x,x^{\prime }\right)=\exp\left(-\gamma {||x-x^{\prime }||}^{2}\right),\;\text{for}\;\;\gamma >0, \end{array}$$

Gaussian radial basis function

(11) $$\begin{array}{c} k\left(x,x^{\prime }\right)=\exp\left(-\frac{{||x-x^{\prime }||}^{2}}{2\sigma^{2}}\right) \end{array},$$

hyperbolic tangent

(12) $$\begin{array}{c} k\left(x,x^{\prime }\right)=\tanh\left(\kappa x\cdot x^{\prime }+c\right),\;\text{for some (not all)}\;\kappa >0,\;c<0 \end{array}.$$

The goal of SVM is to produce a model that predicts target values of data instances in the test set for which only the attributes are given. The following decision function is applied to determine which class the sample belongs to:

(13) $$\begin{array}{c} f\left(x\right)=\mathrm{sgn}\left(\overset{l}{\underset{i=1}{\sum }}y_{i}a_{i}^{*}k\left(x_{i},x_{j}\right)+b^{*}\right) \end{array}.$$

The parameters *a*_i_* and b* are the optimum solutions for specificity.

### 2.4. Clinical Data

Qi-deficient patients, based on TCM theory and clinical practice, exhibit the following characteristics: dispirited spirit, lack of qi and no desire to speak, discouraged, small voice; giddy dazzled, palpitations, sweaty, qualitatively weak tongue, tender, and feeble pulse. By contrast, yin-deficient patients are characterised as follows: emaciation, feverish sensation over the five centres, hot flushes, night sweats, and dry stool, among others. The subjects comprised voice signals from people of different age and sex. The detailed information is listed in Tables 1 and 2.

All these data are collected by our research partner the TCM Syndrome Laboratory of the Shanghai University of Traditional Chinese Medicine in its affiliated hospitals including the Longhua Hospital and the Shuguang Hospital. The voice is recorded using a high-performance microphone (the band is AKG model HSD171) and a 16-bit A/D converter connected to a computer. The frequency response range of the microphone is 60 Hz to 17 kHz. Its sensitivity is 1 mv/Pa (−60 dBV) with an impedance of 600 ohms. In addition, the sample frequency is 16 kHz. All the voice samples were collected by the acquisition system developed based on Visual C++ 6.0. The endpoint detection algorithm was applied to remove the nonvoice portions of the leading and trailing of each utterance.

**Figure 3:**
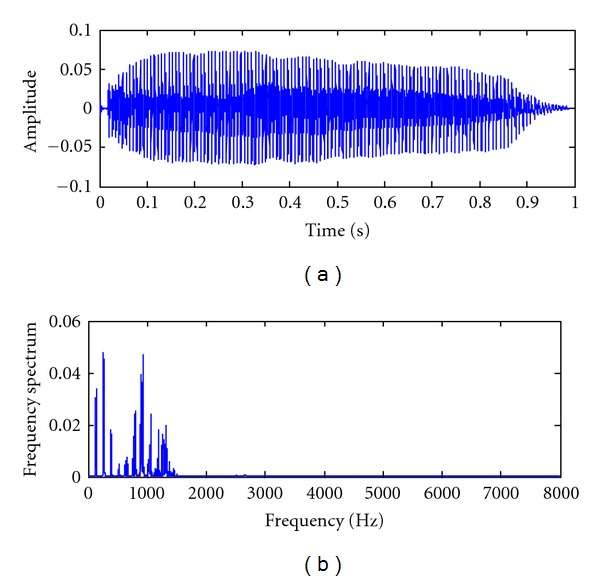
Original signal and amplitude spectrum for it.

The vowel /a/ was chosen as the utterance. Each subject produced a stable phonation of a sustained English vowel /a/ lasting about one second. This vowel is chosen because both patients and healthy subjects can easily pronounce this vowel. In addition, the vocal organ is not abuttal, and there is no obstacle in the cavity when this vowel is pronounced [20]. The pronunciation flow is unblocked, and a periodical waveform can be produced. Therefore, the vowel /a/ was mainly recently chosen as the utterance. The time-domain plot and spectrum of the vowel /a/ are shown in Figure 3.

### 2.5. Processing of Voice Signal Using WPT

The voice signals including three kinds of samples were analyzed using WPT in the first stage of processing of sample identification. Five levels of wavelet packet decomposition were applied as the preprocessing step for all subjects. The maximum frequency in high-frequency bands of the original signal is 8 kHz under the sample frequency 16 kHz, then the frequency interval of the coefficients for the frequency bands is 250 Hz in fifth level.

### 2.6. The SampEn Computation

**Figure 4:**
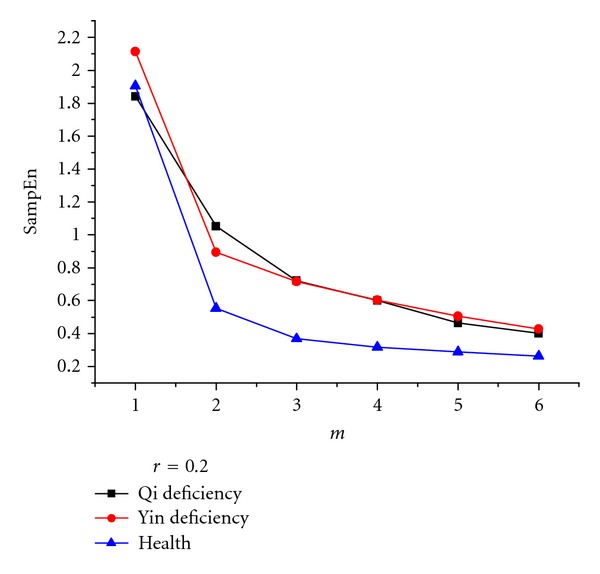
Influence of *m* on the separability among three classes using SampEn. The maximum separability is achieved with *m* = 2.

**Figure 5:**
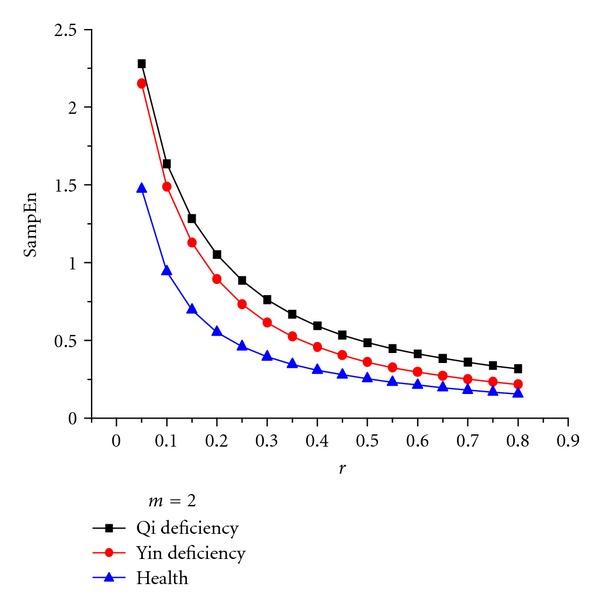
Influence of *r* on the separability among three classes using SampEn. The maximum separability is achieved with *r* = 0.1 ~ 0.25.

In the second stage, SampEn values of approximation and detailed coefficients at each level of the wavelet decomposition were computed for the voice signals of the healthy subjects, as well as yin- and qi-deficient patients. In choosing the optimum parameters *m* and *r*, Pincus suggested *m* = 2 and *r* = 0.1  *δ* to 0.25 *δ*, where *δ* is the standard deviation of the original signal *u*(*i*), *i* = 1,…, *N*. One of the original signals was chosen and analysed using different *m* and *r* values to better illustrate the advantages of the choice. The results are shown in Figures 4 and 5. We can easily see that the difference in the SampEn values was the largest among the signals of the three kinds of samples (shown in Figure 5). This condition indicates that the choice of the value *m* = 2 is appropriate. We can also see that the SampEn value decreased as the parameter increased, although in a lower degree. Therefore, *r* is selected as 0.2 *δ* appropriately.

## 3. Results and Discussion

### 3.1. Results on SampEn Values for WPT Coefficients

Voice signals from qi- and yin-deficient, as well as healthy, subjects were decomposed into sub-bands using WPT. The frequency bands for these sub-bands were as follows: *S*_−1_^*n*^ (the frequency interval is 4 kHz, *n* = 0, 1), *S*_−2_^*n*^ (the frequency interval is 2 kHz, *n* = 0, 1,2, 3), *S*_−3_^*n*^ (the frequency interval is 1 kHz, *n* = 0, 1,2,…, 7), *S*_−4_^*n*^ (the frequency interval is 0.5 kHz, *n* = 0, 1,2,…, 15), and *S*_−5_^*n*^ (the frequency interval is 0.25 kHz, *n* = 0, 1,2,…, 31). SampEn values of the approximated and detailed coefficients under fifth-level WPT decompositions were computed using the selected parameters in Section 2.6.

**Figure 6:**
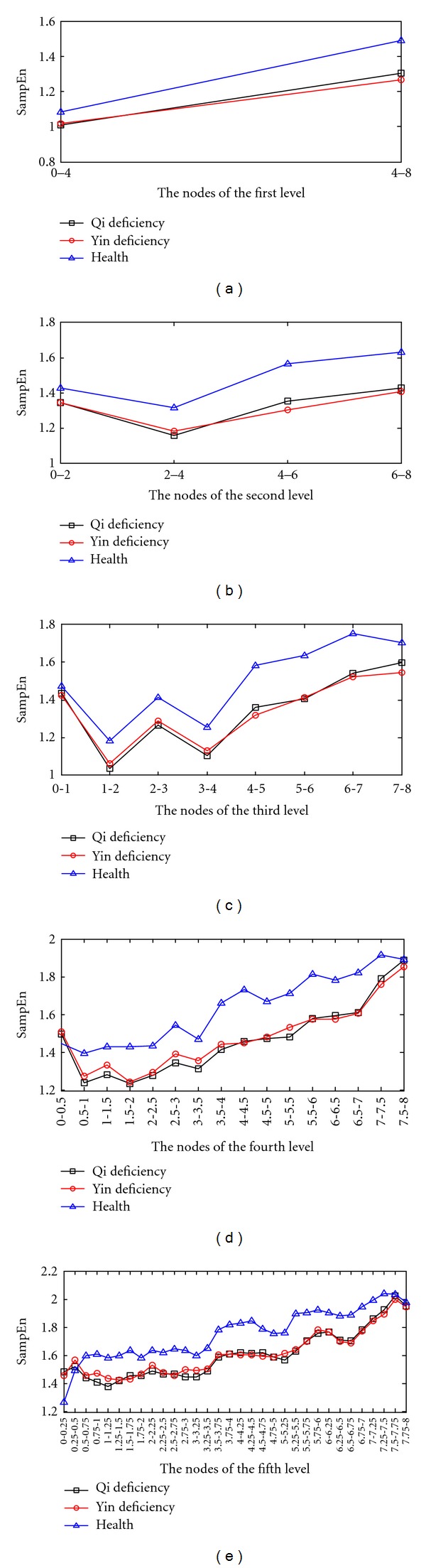
The SampEn values for the coefficients of WPT: (a)–(e) SampEn values for the first to the fifth level coefficients.

The average SampEn values for the coefficients of the 1–5 levels are illustrated in Figures 6(a)–6(e). The differences between healthy and qi- or yin-deficient samples are relatively high, except in 0–0.5 kHz and 7.5–8 kHz of the forth level and 0.25–0.0.5 kHz, 7.5–7.75 kHz and 7.75–8 kHz of fifth level. However, the differences between the qi- and yin-deficient samples are relatively low apart from the following frequency ranges: 0 kHz to 8 kHz in the 1–5 levels.

We also can see in Figures 6(a)–6(e) that, with increasing wavelet packet levels, the frequency bands become more subtle. At the same time, more feature information contained in the voice signal is represented. Slight changes that cannot be reflected in low scales will be represented in high scales. Furthermore, the overall trend of SampEn values for qi-deficient, yin-deficient and healthy samples tends to be higher as frequency increases. The SampEn values of qi-deficient samples are lower than those of yin-deficient samples in most of frequency bands of 0–4 kHz in 1–5 levels, while the SampEn values for qi- and yin-deficient samples are intertwined in 4–8 kHz.

### 3.2. Statistical Analysis

Statistical analysis software, SPSS 20, was applied to analyse the differences among the samples. All SampEn values of the WPT coefficients from the first to the fifth levels were analyzed to obtain the features with significant differences among the three groups of samples. Tables 3, 4, and 5 shows there were 47 frequency bands having SampEn values with significant differences from 1 to 5 level.

### 3.3. Classification Analysis

LibSVM 2.93 was used to identify the auscultation signal. The feature parameters with remarkable differences (47 features in different bands) were chosen as the input vectors consistent with the format of the LibSVM. The SVM type is C-SVC, and the RBF function was chosen as the kernel function for nonlinear training and testing after numerous experiments. The optimum parameters *c* and *g* were obtained as 0.25 and 0.0625 using cross-validation (*c* is the penalty factor, and *g* is the parameter for kernel function).  Table 6 shows the classification results using SVM, in which a good result for classifying the samples (up to 96%) was obtained. This finding proves that the method applied in this paper is impressive.

### 3.4. Discussion

The quantitative analysis of the speech of healthy persons and deficient patients is one of the important task in the objectification and modernization of auscultation of TCM. The voices of healthy people are natural, gentle, clear, fluent, and understandable, while the patients with deficient syndrome speak feebly in low voice and discontinuously. The SampEn values of healthy samples are higher than qi- or yin-deficient samples in most of frequency bands. It may demonstrate that healthy persons have more physiological adaptabilities than the patients with deficiency syndrome. The variation trend of the SampEn values in the qi- and yin-deficient samples were almost similar, perhaps because both qi- and yin-deficient subjects belong to the deficiency syndrome, and the differences of voice signal characteristic between them are not remarkably significant. The classification result demonstrated that the SVM classifier was effective for the identification of the auscultation signals. Therefore auscultation analysis based on WPT-SampEn-SVM was suitable for the identification among qi- and yin-deficient, as well as healthy, subjects.

## 4. Conclusions

In this paper, we proposed a new method in identifying the auscultation signals in TCM including three kinds of samples, namely, qi- and yin-deficient, as well as healthy, samples. Instead of solely using traditional time or frequency domain features, we applied nonlinear dynamic parameter SampEn together with time and frequency analysis method to come up with the wavelet packet to obtain our feature parameters. Wavelet packets are specifically used because of their capability to partition both low- and high-frequency signals. At the same time, SampEn, a statistics parameter used to measure the predictability of the current amplitude values of a physiological signal, is adopted in our research to analyze the signals from three kinds of samples. Experimental results illustrated that WPT-SampEn-SVM-based analysis was suitable for the identification among qi- and yin-deficient, as well as healthy, subjects. Our future research will improve the performance of indentifying deficient patients by analyzing the SampEn variability of the signals of reconstructed coefficients in different frequency bands of each level. In addition, the clinical sample size will be extended for the verification of our methods.

## Figures and Tables

**Table 1:** The groups and sex of all samples in the experiments.

	Healthy	Qi deficiency	Yin deficiency	Head count
Sample number	27	116	38	181
Man	9	39	11	59
Woman	18	77	27	122

**Table 2:** The ages of three groups' samples in the experiments.

Age (year)			
	Healthy	Qi deficiency	Yin deficiency
Max. age	54	76	80
Min. age	19	6	18
Average age	24.9	42.4	52.1

**Table 3:** SampEn values for the subbands' coefficients in first, second and third levels with significant differences.

Frequency band (kHz)	Qi deficiency		Yin deficiency		Healthy		*P* value
	Mean	SD	mean	SD	Mean	SD	
4–8	1.303	0.346	1.266	0.346	1.490	0.374	0.011
4–6	1.356	0.356	1.304	0.358	1.567	0.357	0.005
6–8	1.428	0.312	1.410	0.289	1.632	0.220	0.002
3-4	1.104	0.266	1.131	0.306	1.256	0.296	0.035
4-5	1.359	0.364	1.319	0.406	1.582	0.362	0.005
5-6	1.406	0.334	1.412	0.339	1.636	0.332	0.003
6-7	1.541	0.292	1.524	0.313	1.755	0.251	0.001
7-8	1.600	0.266	1.544	0.279	1.703	0.269	0.044

**Table 4:** SampEn values for the subbands' coefficients in the fourth level with significant differences.

Frequency band (kHz)	Qi deficiency		Yin deficiency		Healthy		* P *value
	mean	SD	mean	SD	mean	SD	
0.5–1	1.240	0.220	1.276	0.289	1.397	0.293	0.005
1–1.5	1.284	0.263	1.334	0.310	1.434	0.324	0.029
1.5–2	1.238	0.307	1.246	0.352	1.431	0.303	0.021
2.5–3	1.346	0.290	1.392	0.342	1.544	0.287	0.009
3–3.5	1.317	0.277	1.359	0.325	1.472	0.306	0.048
3.5–4	1.418	0.348	1.444	0.355	1.661	0.351	0.002
4–4.5	1.459	0.373	1.452	0.406	1.733	0.358	0.001
4.5–5	1.476	0.351	1.481	0.385	1.670	0.386	0.014
5–5.5	1.482	0.337	1.532	0.346	1.712	0.313	0.004
5.5–6	1.582	0.284	1.578	0.329	1.815	0.283	0.001
6–6.5	1.596	0.296	1.576	0.335	1.782	0.306	0.005
6.5–7	1.610	0.274	1.608	0.321	1.824	0.224	0.002
7–7.5	1.793	0.232	1.759	0.242	1.914	0.219	0.015

**Table 5:** SampEn values for the subbands' coefficients in fifth level with significant differences.

Frequency band (kHz)	Qi deficiency		Yin deficiency		Healthy		*P* value
	mean	SD	mean	SD	mean	SD	
0.00–0.25	1.487	0.304	1.459	0.275	1.269	0.386	0.020
0.50–0.75	1.446	0.242	1.457	0.250	1.601	0.238	0.008
0.75–1.00	1.410	0.273	1.475	0.326	1.612	0.321	0.004
1.00–1.25	1.380	0.295	1.436	0.327	1.584	0.323	0.005
1.25–1.50	1.423	0.301	1.429	0.344	1.603	0.374	0.025
1.50–1.75	1.459	0.323	1.433	0.378	1.635	0.322	0.041
2.50–2.75	1.470	0.302	1.459	0.366	1.647	0.328	0.020
2.75–3.00	1.448	0.306	1.501	0.358	1.638	0.286	0.015
3.25–3.50	1.489	0.287	1.508	0.323	1.652	0.315	0.025
3.50–3.75	1.587	0.335	1.608	0.342	1.784	0.336	0.008
3.75–4.00	1.611	0.383	1.613	0.367	1.823	0.354	0.007
4.00–4.25	1.622	0.379	1.607	0.371	1.832	0.387	0.005
4.25–4.50	1.617	0.349	1.605	0.369	1.847	0.333	0.002
4.50–4.75	1.624	0.321	1.594	0.381	1.791	0.346	0.016
4.75–5.00	1.588	0.337	1.588	0.377	1.759	0.359	0.028
5.00–5.25	1.567	0.346	1.614	0.353	1.761	0.332	0.008
5.25–5.50	1.631	0.300	1.642	0.355	1.897	0.216	0.000
5.50–5.75	1.703	0.277	1.700	0.315	1.903	0.254	0.002
5.75–6.00	1.760	0.255	1.784	0.266	1.924	0.205	0.005
6.00–6.25	1.767	0.268	1.767	0.278	1.902	0.245	0.020
6.25–6.50	1.712	0.272	1.702	0.336	1.884	0.257	0.006
6.50–6.75	1.706	0.287	1.690	0.349	1.891	0.242	0.005
6.75–7.00	1.783	0.252	1.776	0.305	1.945	0.223	0.007
7.00–7.25	1.863	0.230	1.848	0.251	1.991	0.193	0.018
7.25–7.50	1.923	0.223	1.896	0.249	2.040	0.161	0.010
7.50–7.75	2.026	0.164	1.997	0.149	2.034	0.251	0.037

**Table 6:** Prediction accuracies using SVM.

Group numbers	Accuracy for each class	Overall accuracy
Qi deficiency	99%	96%
Yin deficiency	89%	
Healthy	93%	
